# Proteome and secretome profiling of zinc availability in *Cryptococcus neoformans* identifies Wos2 as a subtle influencer of fungal virulence determinants

**DOI:** 10.1186/s12866-021-02410-z

**Published:** 2021-12-13

**Authors:** B. Ball, E. Woroszchuk, A. Sukumaran, H. West, A. Afaq, D. Carruthers-Lay, B. Muselius, L. Gee, M. Langille, S. Pladwig, S. Kazi, A. Hendriks, J. Geddes-McAlister

**Affiliations:** Department of Molecular and Cellular Biology, University of Guelph, 50 Stone Rd. E, Guelph, Ontario N1G 2W1 Canada

**Keywords:** *Cryptococcus neoformans*, Fungal pathogenesis, Zinc limitation, Quantitative proteomics, Polysaccharide capsule, Virulence

## Abstract

**Background:**

Fungal infections impact over 25% of the global population. For the opportunistic fungal pathogen, *Cryptococcus neoformans*, infection leads to cryptococcosis. In the presence of the host, disease is enabled by elaboration of sophisticated virulence determinants, including polysaccharide capsule, melanin, thermotolerance, and extracellular enzymes. Conversely, the host protects itself from fungal invasion by regulating and sequestering transition metals (e.g., iron, zinc, copper) important for microbial growth and survival.

**Results:**

Here, we explore the intricate relationship between zinc availability and fungal virulence via mass spectrometry-based quantitative proteomics. We observe a core proteome along with a distinct zinc-regulated protein-level signature demonstrating a shift away from transport and ion binding under zinc-replete conditions towards transcription and metal acquisition under zinc-limited conditions. In addition, we revealed a novel connection among zinc availability, thermotolerance, as well as capsule and melanin production through the detection of a Wos2 ortholog in the secretome under replete conditions.

**Conclusions:**

Overall, we provide new biological insight into cellular remodeling at the protein level of *C. neoformans* under regulated zinc conditions and uncover a novel connection between zinc homeostasis and fungal virulence determinants.

**Supplementary Information:**

The online version contains supplementary material available at 10.1186/s12866-021-02410-z.

## Background

Fungal diseases are critical burdens on the global healthcare system, in which fungal infections inflict disease on 25% of the world’s population. Furthermore, the frequency of invasive fungal infections is rapidly increasing due to growing numbers of immunocompromised individuals [1, 2]. This detrimental influence on human health has ignited a growing field of research into understanding these understudied and underdiagnosed pathogens. The human fungal pathogen, *Cryptococcus neoformans,* is a severe clinical threat responsible for cryptococcosis, which may manifest as meningitis or meningoencephalitis [3]. Treatment options against the pathogen are limited due to a lack of available drugs because of host toxicity, difficulty in development due to eukaryotic homology, and the growing concern of emergence and evolution of resistant strains [4–6]. *C. neoformans* is an opportunistic fungus found ubiquitously within the environment and is equipped with sophisticated virulence determinants, including a polysaccharide capsule, melanin, thermotolerance, and extracellular enzymes [7]. This arsenal of determinants provides resources to modulate the host immune system and promote fungal survival in over 230,000 immunocompromised individuals (e.g., HIV/AIDS patients) annually [8, 9]. To overcome the expanding dangers of serious fungal infections, it is crucial to better understand the fungi-specific mechanisms required to infect and survive within a human host.

Transition metals, including iron, copper, and zinc, are fundamental requirements for the survival of all living organisms [10]. The concept of transition metal homeostasis is a well-established research discipline concerning pathogenic microbes. The connection between virulence and trace elements of transition metals is dominated mainly by the study of iron limitation involving acquisition, storage, and detoxification [11–17]. However, beyond the iron paradigm, zinc is an essential cofactor of many proteins providing pivotal catalytic and structural roles; zinc-binding proteins constitute approximately 9% of the eukaryote and 5–6% of the prokaryote proteome [18]. Despite its inactive redox status, excess zinc induces cellular toxicity and physiological stress, which includes protein inhibition from binding unfavourably to sites not normally associated with a metal ion [19–22]. Additionally, previous studies report the impediment of fungal growth under reduced zinc conditions in minimal and chelated mediums [23]. Therefore, it is necessary to tightly regulate intracellular labile zinc content to maintain precise metal homeostasis. Mammalian hosts manipulate these delicate margins of nutritional requirements as a sophisticated technique to combat pathogenic microbes from securing essential micronutrients. Moreover, host cells may also release transition metals targeted towards invading microbes to lethal or growth-inhibiting concentrations. These collective protective measures have been termed ‘nutritional immunity’ [24–26].

Pathogenic microorganisms have evolved counterattack measures to facilitate zinc assimilation and ensure survival within the host. We recently explored the impact of zinc availability in the bacterial pathogen, *Klebsiella pneumoniae* through proteomic profiling and uncovered a novel connection between zinc homeostasis and capsule regulation [22]. For fungal pathogens, strategies to maintain zinc homeostasis are best characterized in the model organism *Saccharomyces cerevisiae,* in which zinc assimilation in zinc-limiting conditions is mainly allocated by the Zrt-Irt-like (Zip) protein family of membrane transporters [27, 28]. In addition, *C. neoformans ZIP1* deficient mutants demonstrated zinc limited growth defects, reduced infectivity towards host immune cells, and impaired virulence in a murine model of cryptococcal infection [29]. Conversely, cation diffusion facilitator proteins are involved in processes of zinc-excess (or replete) conditions and mediate intracellular zinc transport to organelles for storage or detoxification, suggesting homeostasis of microbial and environmental zinc levels is tightly regulated through protein activation and cellular remodeling [30]. Moreover, heat shock proteins (HSPs), including Wos2 (P21), a homolog of P23 in *Schizosaccharomyces pombe*, are involved in cell cycle progression and show a decrease in expression when cells enter stationary growth or are grown under nutrient limited conditions [31, 32]. These findings demonstrate a connection between cell cycle progression and metal ion homeostasis. Given the crucial role of master regulators (e.g., Zap1, a zinc finger transcription factor) of metal ion homeostasis in biological processes of fungal pathogens, there is a need to comprehensively define zinc metabolism determinants in relation to fungal survival and development [33, 34]. Mass spectrometry-based proteomics is a technology capable of defining changes in cellular remodeling of a pathogen under distinct growth conditions, and detecting proteins critical to microbial adaptation [35, 36].

**Fig. 1:**
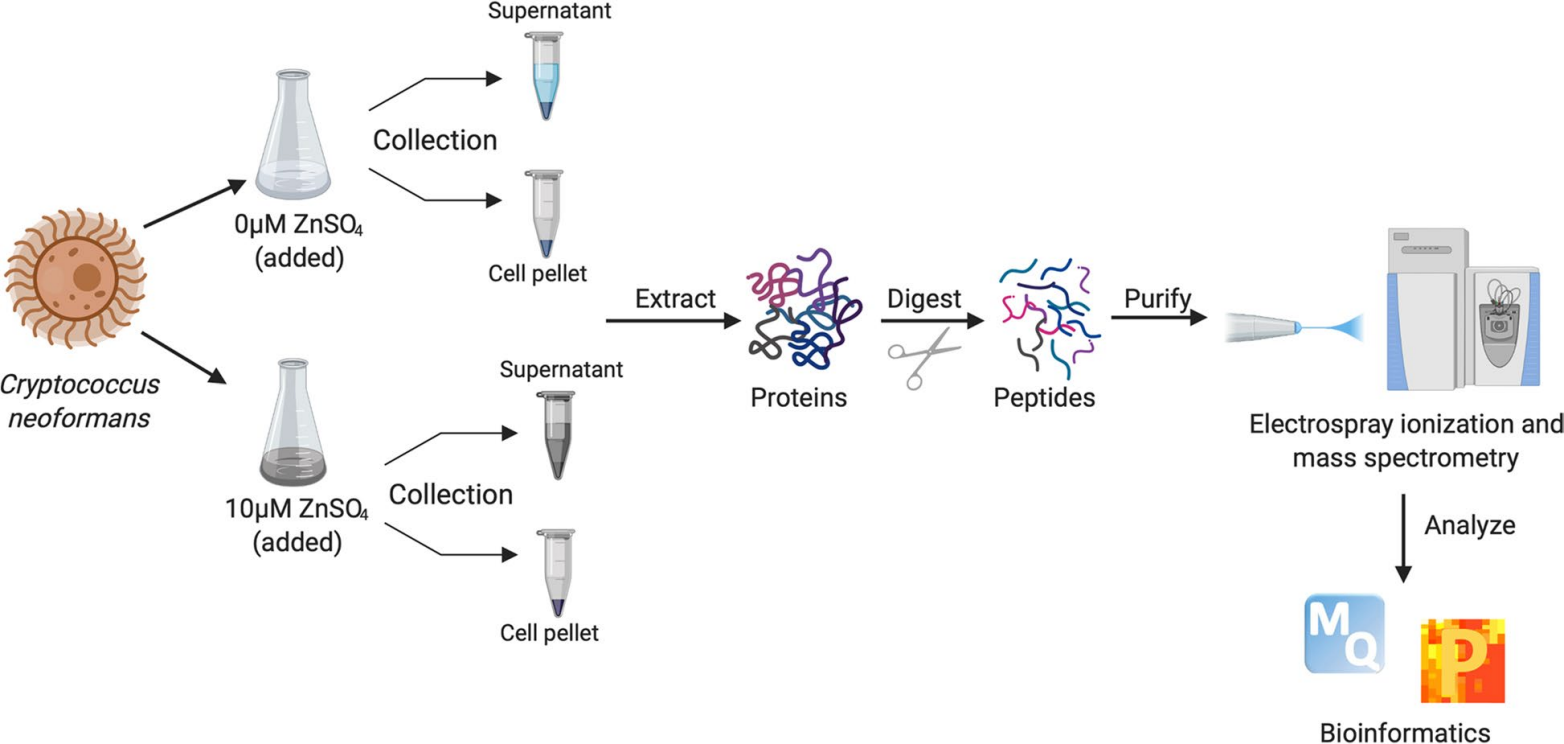
Overview of experimental design and mass spectrometry workflow. Cell pellets and supernatant from *C. neoformans* cultured in minimal medium (MM) MM + Zn or MM-Zn were subjected to protein extraction (e.g., sonication and detergent) and enzyme digestion (e.g., trypsin/Lys-C) followed by purification (C18 STAGE-tips) and measurement on a quadrupole orbitrap mass spectrometer [37–40]. Data analysis and visualization performed with MaxQuant and Perseus. Experiment performed in biological quadruplicate. Figure generated with Biorender.com

Here, we describe the first quantitative proteomics investigation focused on how a zinc-limited environment modulates the cellular proteome and secretome of *C. neoformans* (Fig. 1)*.* A comparative analysis of the physiological responses of fungal proliferation in zinc-limited and -replete conditions identified differentially abundant proteins. Investigation of proteins produced during zinc starvation revealed a shift of the signature proteomic profile towards transcription and metal acquisition, including a significant increase in abundance for the zinc transporter, *ZIP1*. Conversely, we detected a Wos2 protein (a HSP90 co-chaperone) at high abundance within the extracellular environment under replete conditions. Deletion of *WOS2* (CNAG_07558) revealed subtle phenotypic changes of fungal thermotolerance, zinc utilization, melanin production*,* and capsule elaboration. These data suggest a novel connection between regulation of zinc homeostasis and fungal virulence determinants at the protein level. Overall, this study provides crucial details to bridge the gaps in knowledge surrounding the global impact of zinc deprivation in the pathogen *C. neoformans.*

## Results

### Modulating zinc availability promotes cellular remodeling of the *C. neoformans* proteome

To explore the relationship between zinc availability and protein production in *C. neoformans*, we profiled the cellular proteome (cell pellet) and secretome (extracellular environment) under zinc-limited and -replete conditions using mass spectrometry-based proteomics (Fig. 1). We hypothesized that changes to zinc availability in the culture medium would influence the production of proteins to promote fungal survival and adaptation. This information drives our understanding of fungal response to changing environmental conditions and uncovers proteins critical to cellular remodeling for an effective response.

**Fig. 2:**
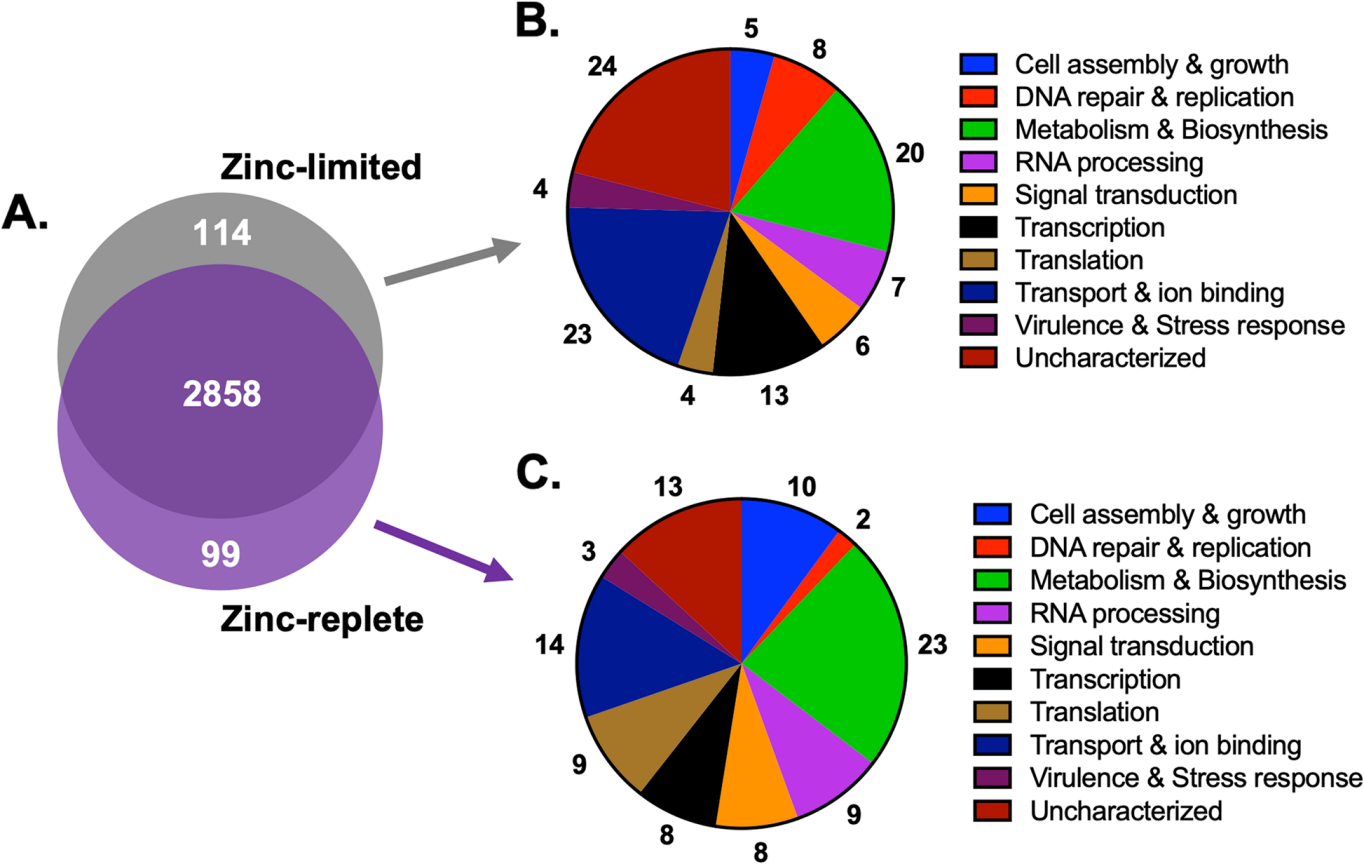
Remodeling of the *C. neoformans* zinc-regulated cellular proteome. **A** Venn diagram for number of unique proteins identified in the cellular proteome under zinc-limited (114; grey) and zinc-replete (99; purple) conditions with 2858 proteins commonly identified. **B** Distribution of Gene Ontology Biological Processes for all proteins identified in the zinc-limited cellular proteome. **C** Distribution of Gene Ontology Biological Processes for all proteins identified in the zinc-replete cellular proteome. Experiment performed in biological quadruplicate

Within the cell pellet, we detected and quantified 3411 unique proteins (3071 proteins after valid value filtering) across the samples, representing 46% of the proteome of *C. neoformans*. Of these, 2858 proteins were identified under both zinc-limited and -replete conditions, designated as a core proteome, whereas 114 proteins were detected only under zinc-limited conditions and 99 proteins were detected only under zinc-replete conditions (Fig. 2A). Of the unique proteins under zinc-limited conditions, we observed the largest categories of proteins associated with ‘metabolism and biosynthesis’, ‘transport and ion binding’, and ‘uncharacterized’ (Fig. 2B). These were also well-represented categories under zinc-replete conditions, but to a lesser extent for ‘transport and ion binding’ and ‘uncharacterized’ compared to a greater emphasis on ‘translation’ as well as ‘cell assembly and growth’ (Fig. 2C). Taken together, this qualitative assessment highlights a zinc-regulated protein signature within the cellular proteome of *C. neoformans*.

**Fig. 3:**
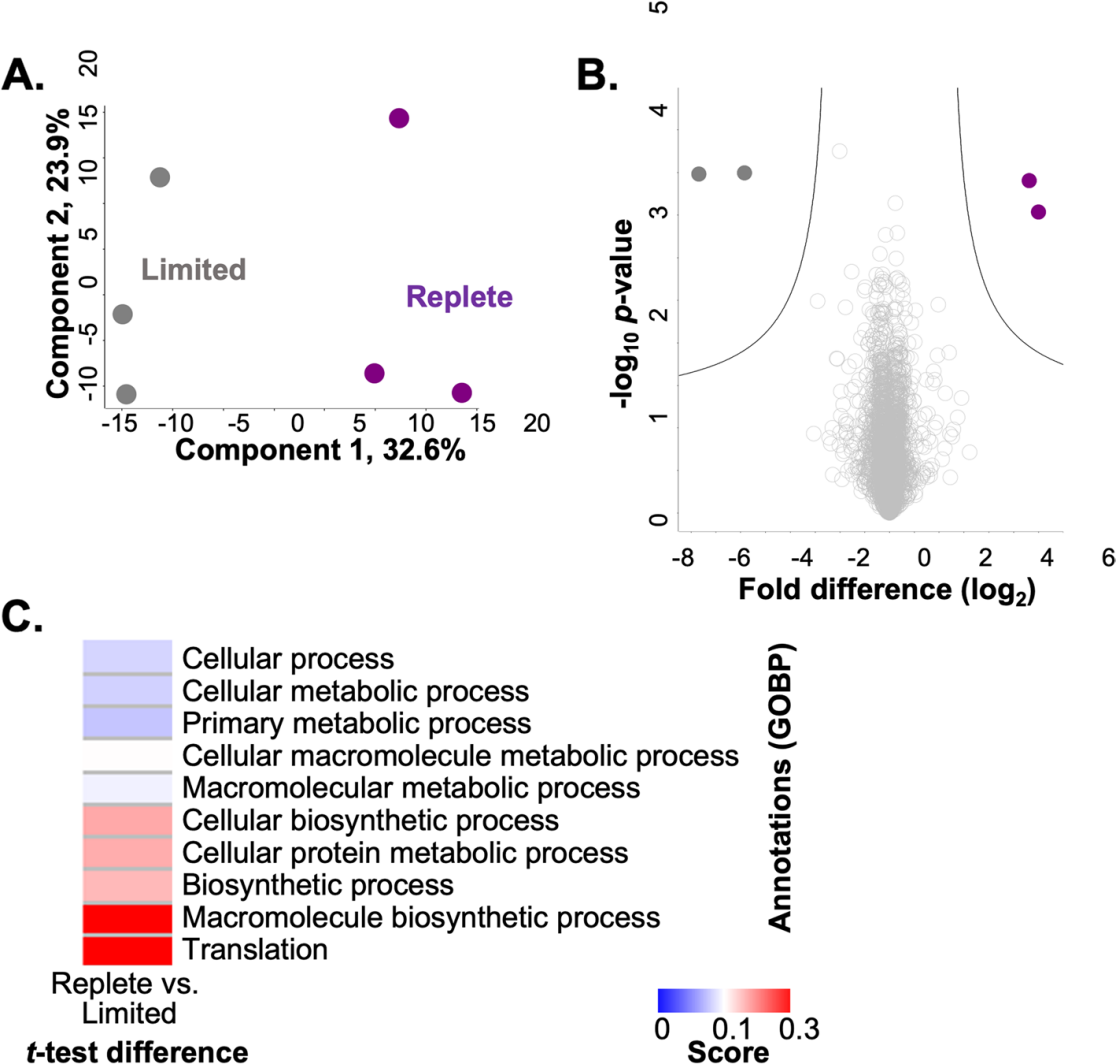
Cellular proteome profiling of zinc limitation in *C. neoformans*. **A** PCA plot of *C. neoformans* biological replicates under limited (grey) and replete (purple) conditions; clustering based on growth conditions (component 1) and biological variability (component 2). **B** Volcano plot depicting all proteins identified under zinc-limited and -replete (10 μM) conditions, highlighting proteins with significant increases or decreases in abundance during limited (grey) and replete (purple) conditions. Student’s *t*-test, *p-value* ≤ 0.05; FDR = 0.05; S0 = 1. **C** 1D annotation enrichment based on Gene Ontology Biological Processes (Student’s *t*-test, *p-value* ≤ 0.05; FDR = 0.05; − 0.5 < score < 0.5). Experiment performed in biological triplicate

To assess factors influencing the proteomic profiling, we performed a principal component analysis (PCA), which indicated separation between zinc-limited and -replete conditions as the largest component of distinction (component 1, 32.6%), while biological variability amongst the replicates accounted for the second most impactful component (component 2, 23.9%) (Fig. 3A). Biological replicate reproducibility was 96.8–97.0%. (Supp. Fig. 1). Next, we analyzed the proteomics data to detect proteins with significant changes in abundance under the different zinc conditions to identify specific proteins impacted by modified zinc availability (Fig. 3B). Under zinc-limited conditions, we observed a significant increase (*p*-value ≤0.05; FDR = 0.05) in abundance of a zinc transporter, *ZIP1* (CNAG_00895; > 5-fold), aligning with previously reported transcript profiling of zinc limitation in *C. neoformans* [29]. This finding supports our experimental design and ability to profile zinc-regulated changes in the fungal proteome. We also detected a cobalamin synthesis protein (CNAG_02548; > 7-fold) with higher abundance under limited conditions, which builds upon a known relationship between cobalamin biosynthesis and zinc in microbial systems [41]. On the contrary, under replete conditions, we observed a significant increase in abundance of a ribosomal protein (CNAG_03127; > 5-fold), supporting our observation of translation-associated proteins identified under replete conditions, and a hypothetical protein (CNAG_01290; 6-fold) with no known orthologs.

To define a comprehensive impact of zinc availability on fungal cells by considering the abundance of all proteins (and not only those with significant differences in abundance) we performed a 1D annotation enrichment (i.e., tests for every annotation term whether the corresponding numerical values have a preference to be systematically larger or smaller than the global distribution of the values for all proteins [42]) based on Gene Ontology Biological Processes (Fig. 3C). Here, we observed the greatest enrichment of proteins associated with ‘translation’ and ‘macromolecule biosynthetic process’ under zinc-replete conditions, compared to ‘cellular process’ and ‘cellular and primary metabolic process’ under zinc-limited conditions. Taken together, enrichment of cellular and metabolic processes under zinc-limited conditions may be associated with intrinsic responses to a nutrient-poor environment. However, our assessment of specific proteins with altered abundance between the tested growth conditions teases apart nutrient-limited vs. zinc-specific responses, demonstrating cellular remodeling at the protein level influenced by zinc availability.

### Zinc availability alters secreted protein profiles

**Fig. 4:**
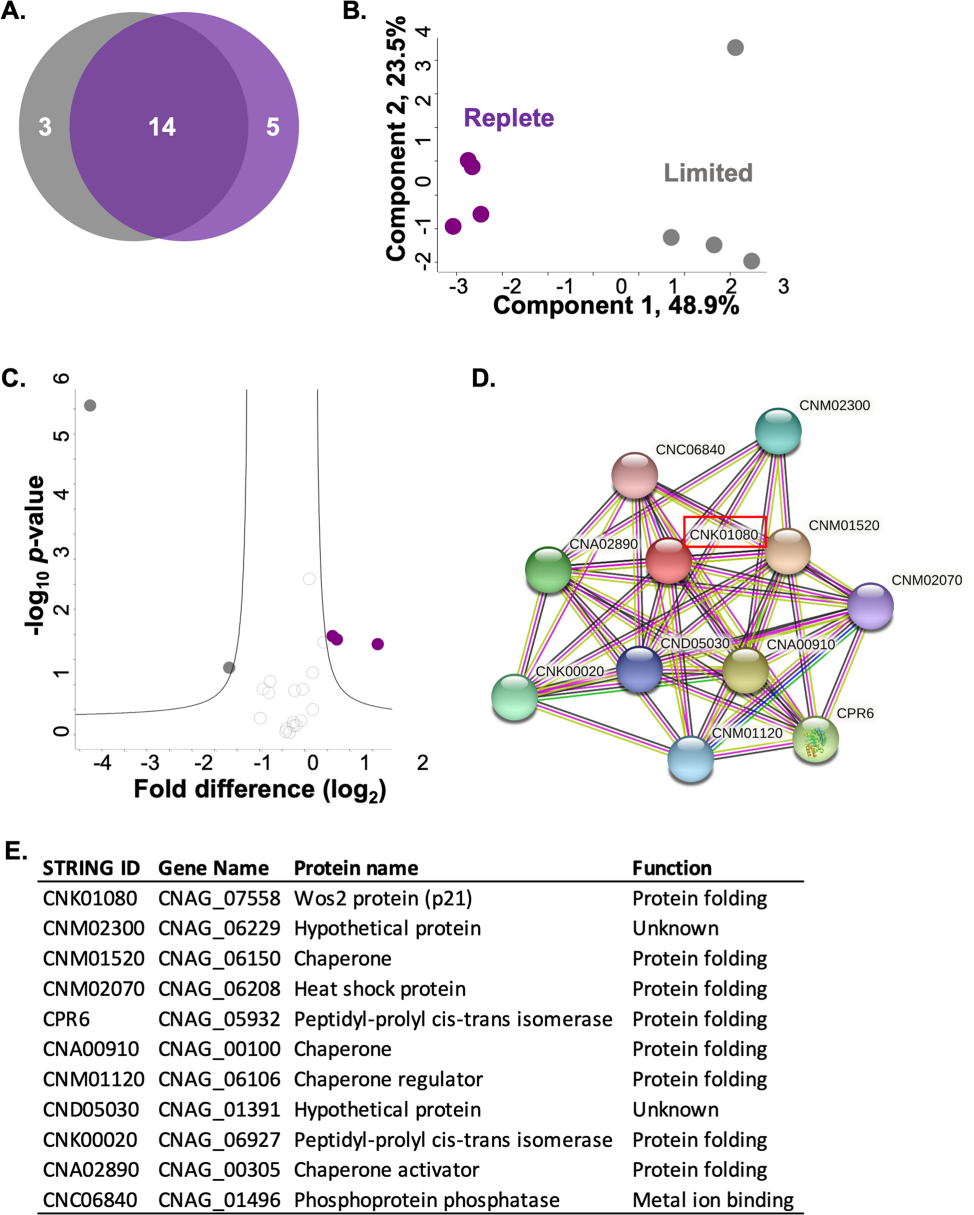
Secretome profiling of zinc limitation in *C. neoformans*. **A** Venn diagram for number of unique proteins identified in the secretome under zinc-limited (3; grey) and zinc-replete (5; purple) conditions with 14 proteins commonly identified. **B** PCA plot of *C. neoformans* biological replicates under limited (grey) and replete (purple) conditions; clustering based on growth conditions (component 1) and biological variability (component 2). **C** Volcano plot depicting all proteins identified under zinc-limited and -replete (10 μM) conditions, highlighting proteins with significant increases or decreases in abundance during limited (grey) and replete (purple) conditions. Student’s *t*-test, *p-value* ≤ 0.05; FDR = 0.05; S0 = 1. **D** STRING network analysis of predicted and known interacting partners of significantly increased Wos2 (CNK01080; highlighted in red) under replete conditions. **E** STRING interacting partners of Wos2, including protein name and function. Experiment performed in biological quadruplicate

The extracellular environment plays an important role in influencing the secretion and/or release of proteins for nutrient sensing and acquisition in biological systems [43]. Here, we profiled the supernatant (i.e., extracellular environment) of *C. neoformans* grown in zinc-limited vs. -replete conditions to evaluate the role of zinc on fungal secretion. We identified 33 proteins (22 proteins after valid value filtering), of which, 14 were common between the conditions, with three proteins unique to zinc-limited conditions and five proteins unique to zinc-replete conditions (Fig. 4A). A PCA plot demonstrated the largest component of separation between the data sets to be zinc-regulated (component 1, 48.9%) and the second component associated with biological variability (component 2, 23.5%) (Fig. 4B). Biological replicate reproducibility was 83.6–89.4%, demonstrating good reproducibility among the supernatant samples. (Supp. Fig. 2).

Investigation into changes in protein abundance influenced by zinc availability revealed two proteins with a significant increase in abundance (*p*-value ≤0.05; FDR = 0.05) under limited conditions, including actin (CNAG_00483; > 4-fold) and ATP-citrate synthase (CNAG_04640; > 1-fold) (Fig. 4C). Detection of these proteins under zinc-regulation in fungal pathogens is supported by previous work in *Paracoccidioides* [44]. Conversely, three proteins showed a significant increase in abundance under replete conditions, including a hypothetical protein (CNAG_07558; > 1-fold), a HSP60-like protein (CNAG_03891; > 2-fold), and a ribosomal protein (CNAG_04448; > 1-fold). Notably, several proteins identified in the supernatant with traditional intracellular roles (e.g., actin, ribosomal, chaperone) were previously detected in extracellular vesicles of *C. neoformans* upon proteome profiling, conferring a role for these proteins in vesicular transport [45]. Moreover, metabolic, ribosomal, and chaperone proteins have been referred to as ‘moonlighting proteins’ or proteins with changing roles given the environmental conditions. For example, classically intracellular proteins may be produced and released into the environment to assist with colonization and invasion of host cells, as described in other fungal pathogens [46, 47].

### In Silico assessment of zinc-regulated proteins

Based on our proteomic profiling, we identified four proteins with significant changes in protein abundance (two increased in replete; two increased in limited) in the cellular proteome and five proteins with significant changes in abundance (three increased in replete; two increased in limited) in the secretome (Table 1). Given previous reports of Wos2 (P21; P23 homolog in *S. pombe*) showing decreased expression in nutrient-limited conditions [31], and our supporting observation of increased production of Wos2 under nutrient-replete conditions, we further explored the role of this protein in *C. neoformans* (Supp. Fig. 3). Wos2 is a HSP90 co-chaperone associated with capsule growth and cell cycle progression that interacts with additional chaperones and HSP detected within *C. neoformans* vesicles, including CNAG_06150 (CNM01520), as well as metal ion binding proteins (e.g., CNAG_01496; CNC06840) (Fig. 4D; 4E) [31, 32]. The increased abundance of Wos2 under replete conditions suggests a connection between zinc availability and production of virulence determinants in the presence of zinc.

**Table 1:** Proteins with significant changes in abundance from cell pellet (cellular proteome) and supernatant (secretome) profiling of *C. neoformans* H99 under zinc-limited and -replete conditions

				Difference (log_**2**_)^b^	
	Gene name^a^	Protein name	Function	Limited	Replete
**Cellular proteome**					
	CNAG_00895	Solute carrier family 39	Transport	5.84	
	CNAG_01290	Uncharacterized protein	Uncharacterized		6.00
	CNAG_02548	Cobalamin synthesis protein	Biosynthesis	7.69	
	CNAG_03127	Small subunit ribosomal protein S23	Translation		5.63
**Secretome**					
	CNAG_00483	Actin	Structure	4.26	
	CNAG_03891	Hsp60-like protein	Protein refolding		2.12
	CNAG_04448	Ribosomal protein L19	Translation		1.12
	CNAG_04640	ATP-citrate synthase	Biosynthesis	1.17	
	CNAG_07558	CS domain-containing protein (Wos2)	Uncharacterized		1.21

^a^Proteins presented according to numerical order of gene identifiers (i.e., CNAG number)

^b^Values presented as positive relative to each comparison

### Wos2 protein influences fungal virulence

**Fig. 5:**
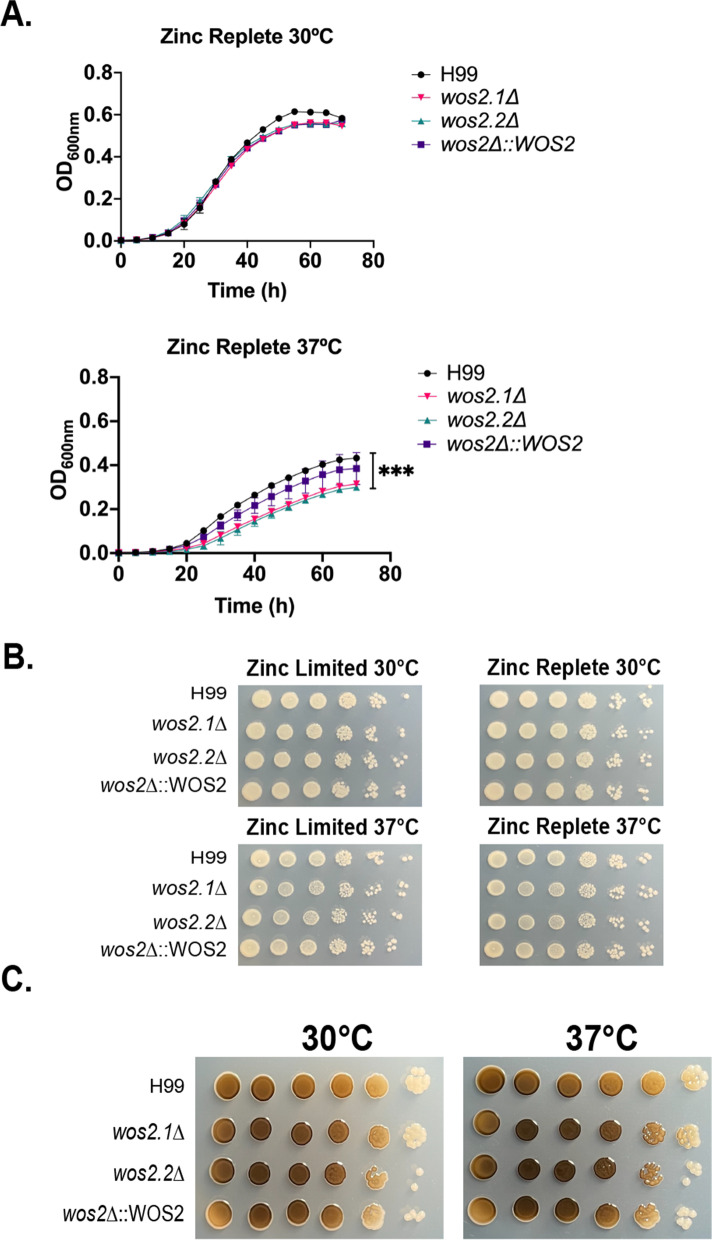
Assessment of thermotolerance, zinc utilization, and melanin production in *C. neoformans*. **A** Growth curve of WT, *wos2*Δ, and *wos2*Δ::WOS2 strains in zinc-replete (MM+ Zn 10 μM) at 30 °C and 37 °C. Growth curves measured at OD_600nm_. **B** Plate dilution assay of WT, *wos2*Δ, and *wos2*Δ::WOS2 strains in zinc-limited and zinc-replete media at 30 °C and 37 °C. Plate assays imaged after 3 d of growth. *Denotes significant difference between comparisons to WT by Student’s *t*-test, asterisks as follows: ***, *P* < 0.005. **C** L-DOPA plates for melanin assay at 30 °C and 37 °C for WT, *wos2*Δ, and *wos2*Δ::WOS2 strains. Plates imaged after 3 d. Experiment performed in biological triplicate and technical duplicate

To further explore the role of Wos2 in *C. neoformans*, we constructed and characterized a deletion strain for potential roles in fungal virulence; deletion of the gene was confirmed by PCR and whole genome assembly (Supp. Fig. 4). To begin, we assessed a role for Wos2 in thermotolerance by comparing the deletion strains to WT at 30 °C and 37 °C in zinc-replete conditions to promote production of Wos2 in the WT for a true comparison with the mutant strain. We observed similar growth for WT and *wos2*Δ at 30 °C (Fig. 5A). Conversely, we observed a significant decrease in growth of the deletion strains at 37 °C compared to WT (Fig. 5A). Next, we evaluated differences in fungal growth under zinc-limited vs. -replete conditions at 30 °C and 37 °C between the strains and observed an increase in growth in the presence of excess zinc at both temperatures (Fig. 5B). We also evaluated a connection between Wos2 and melanin production and observed a slight increase in melanin production in the deletion strains at 37 °C relative to the WT strain (Fig. 5C). These data support a role for Wos2 in thermotolerance, zinc utilization, and melanin production of *C. neoformans*.

**Fig. 6:**
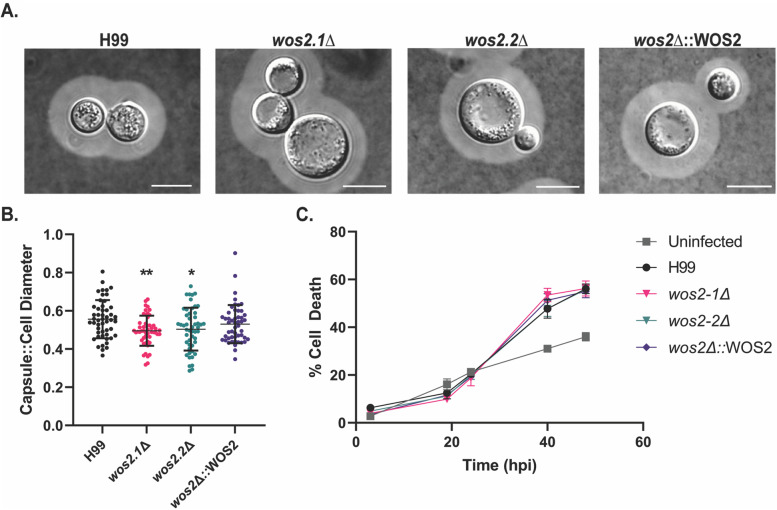
Assessment of capsule production and virulence of *C. neoformans*. **A** Capsule production of WT, *wos2*Δ, and complemented strains measured by India ink staining and differential interference microscopy. Capsule assays performed in LIM at mid-log phase (OD_600nm_ = 1.0–1.2). Scale bar = 4.5 μm. **B** Quantification of the ratio between capsule thickness and cell size diameter for WT, *wos2*Δ, and complemented strains. A minimum of 50 cells were measured for capsule and cell size assays. *denotes significant difference between comparisons to WT by Student’s *t*-test, asterisks as follows: *P < 0.05; **, *P* < 0.01. **C** Quantification of LDH release upon macrophage cell death following co-culture with *C. neoformans* H99, *wos2*Δ, and complemented strains grown in YPD. Uninfected refers to macrophage only culture. Macrophages undergone opsonization with antibody 18b7 prior to co-culture. Experiment performed in biological triplicate and technical duplicate

Given the established connection among Wos2, cell cycle progression, and capsule production [32], we evaluated the impact of deleting *WOS2* from *C. neoformans* H99 on capsule size. Visually, we observed an increase in cell size but a decrease in capsule size in the deletion strains relative to the WT and complemented strains (Fig. 6A). This data was supported by measuring cell diameter and capsule thickness for 50 cells, which showed a significant decrease in the raio of capsule to cell diameter in the *wos2*Δ strains relative to WT and the complemented strains (Fig. 6B). Lastly, based on our observation that deleting *WOS2* from *C. neoformans* influences the production of classical fungal virulence determinants, and the role of capsule production in protecting the fungus from the innate immune response [48], we tested an infection model in immortalized macrophages and measured changes in macrophage cell death over a time course of infection. Here, performing antibody-associated opsonization prior to co-culture of the macrophages with cryptococcal cells, we observed a consistent release of lactate dehydrogenase (LDH), supporting similar patterns of host cell death, among the strains relative to the uninfected control (Fig. 6C). Based on our findings, we propose that WOS2 influences capsule and cell size, but these differences do not correspond to altered infection of macrophages in an in vitro model.

## Discussion

In this study, we use high resolution mass spectrometry-based proteomics to identify and quantify changes in protein abundance within the fungal pathogen, *C. neoformans* under zinc-limited and -replete conditions. Our approach confirms the role of a known zinc transporter (Zip1) in transition-metal acquisition through detection under limited conditions in the cellular proteome and uncovers a new connection between Wos2 (CNAG_07558) and zinc homeostasis within the extracellular environment. Moreover, characterization of the *WOS2* deletion strain demonstrates a subtle role in thermotolerance, zinc utilization, melanin production, and capsule elaboration. Overall, we provide new biological insight into cellular remodeling at the protein level of *C. neoformans* under regulated zinc conditions and uncover a novel connection between zinc homeostasis and regulation of fungal virulence determinants.

Our detection of a HSP90 co-chaperone (CNAG_07558 ortholog) in the supernatant of *C. neoformans* under zinc-replete conditions, in combination with known interactions with vesicle-associated proteins, suggests localization of Wos2 to the vesicular fraction. The connection between vesicle production and zinc homeostasis was previously explored with the introduction of zincosomes [28, 49]. Zincosomes are vesicles containing labile zinc observed in both mammalian and yeast cells, they may serve to both detoxify excess zinc and mobilize the metal upon deprivation; however, the exact nature of these compartments, and the mechanisms of action have not been elucidated [28]. In our dataset, the increased cell size for *wos2*Δ may be associated with a larger vacuole influenced by protein aggregation from a lack of proper folding or degredation and a theory worth exploring further in future studies. We also note that Wos2 or other zinc-associated proteins may assist with detoxification during infection proceeses when zinc may be pumped into the phagosome at high levels to damage engulfed pathogens through intoxification and subsequentely, promote pathogen cell death [50]. Moreover, we identified CNAG_02806 (Zrc1), a critical protein for zinc sequestration and adaptation to zinc excess via vacuole (*C. neoformans*) and zincosomes (*Candida albicans*) in our cellular proteome profiling with a higher abundance under replete conditions (2.09-fold; not significantly different) [51]. This similar trend in increased protein abundance between Zrc1 and Wos2 under replete conditions supports our identification of zinc homeostasis-associated proteins within the extracellular environment of *C. neoformans.* Further exploration into intracellular zinc levels under limited and replete conditions, along with proteomic profiling of intra- and extracellular vesicle contents may provide further evidence of such a regulatory system in *C. neoformans*.

A connection between regulation of zinc transporters (e.g., *ZIP1*, *ZAP1*) and attenuated virulence has been well-defined in *C. neoformans* and *Cryptococcus gattii*, respectively, as well as *C. albicans*, *Aspergillus fumigatus*, and the plant fungal pathogen, *Fusarium oxysporum* [29, 34, 52–54]. In addition, coordinated zinc homeostasis is critical amongst bacterial pathogens for maintaining virulence [22, 55]. These studies underscore the important role zinc plays in microbial pathogenesis and emphasize the outcome on virulence if zinc regulatory mechanisms are altered within the pathogen. However, a link between the production of proteins involved in maintaining zinc homeostasis under nutrient-rich conditions (e.g., zinc replete medium) and virulence has not been explored. Here, we report the novel findings of enhanced production of a virulence determinant (i.e., melanin) for the *wos2*Δ strains relative to WT. Moreover, our observation of increased cell size in the deletion strains may influence interactions with host cells, as defined for cryptococcal titan cells with roles in fungal virulence [56]. Notably, an in vitro macrophage infection model did not highlight changes in virulence; however, we hypothesize that given the subtle difference in virulence determinant regulation, the fungal strains may differ in their ability to infect and potentially, disseminate within an in vivo murine model of infection.

Fungal infections present unique challenges to treatment, including a limited number of effective antifungal agents influenced by host cytotoxicity, intrinsic resistance, and the development and emergence of resistant strains [4–6]. To overcome such limitations and uncover new drug development pipelines, the discovery of novel agents targeting new pathways (e.g., metal ion homeostasis) are being explored [57]. For example, disruption of essential micronutrient (zinc and iron) homeostasis in fungal pathogens by interfering with metal uptake, transcriptional regulation, or sequestration processes in *C. albicans*, established a high-throughput drug screening platform [58]. This work presents a method for identification and verification of new antifungal drugs targeting the perturbation of zinc and iron homeostasis using *C. albicans* as a model fungal pathogen. Given our observation of Wos2 in regulating zinc homeostasis under replete conditions and the influence of *WOS2* on cell size and thermotolerance, we anticipate that the protein may serve as a viable target for therapeutic intervention; however, sensitivity to current antifungals (e.g., azoles) is a logical experimental direction to follow in the future [59].

Lastly, our proteomics profiling identified nine proteins with significant changes in abundance under zinc-limited or -replete conditions. Although, several of these proteins are very well characterized (e.g., CNAG_03127, Ribosomal protein; CNAG_00483, Actin; CNAG_04448, Ribosomal protein; CNAG_04640, ATP-citrate synthase) and/or their role in nutrient limitation is well defined (e.g., CNAG_00895, solute carrier Zip1), other candidates are uncharacterized (e.g., CNAG_01290) and/or their role in zinc homeostasis (e.g., CNAG_02548, cobalamin synthesis; CNAG_03891, Hsp60-like) have yet to be explored. We propose future experimentation to investigate the role of these three proteins in zinc homeostasis and fungal virulence to provide further biological insight into connections between the regulatory systems.

## Conclusion

Quantitative proteomic profiling of zinc limitation in *C. neoformans* supports previously reported transcriptomics datasets through detection of zinc transporters regulated by nutrient limitation. In addition, we uncover an uncharacterized protein (CNAG_07558) orthologous to a Wos2 protein associated with cell cycle progression and capsule production. Characterization of the candidate deletion strain defines new roles for Wos2 in *C. neoformans* virulence determinant production, supporting the protein’s role in maintaining zinc homeostasis under replete conditions. Overall, our findings substantially build upon our knowledge of zinc utilization within the fungal pathogen at the protein level and support further exploration into the connection between zinc availability and fungal virulence in *C. neoformans*.

## Materials and methods

### Fungal strains, growth conditions and media

*Cryptococcus neoformans* var. *grubii* strain H99 (serotype A) was used for all analyses and as a reference strain for mutant construction. The wildtype strain was maintained on yeast peptone dextrose (YPD) medium (2% dextrose, 2% peptone, 1% yeast extract) and all mutant strains were maintained on YPD supplemented with 100 μg/mL nourseothricin (NAT) at 30 °C unless otherwise stated. Zinc minimal media (MM-Zn) was prepared with Chelex® 100-treated (Bio-Rad) dH_2_O containing 29.4 mM KH_2_PO_4_, 10 mM MgSO_4_–7 H_2_O, 13 mM glycine, 3 μM thiamine, 0.27% dextrose, and supplemented with 10 μM ZnSO_4_ (MM + Zn) for replete conditions. MM followed the recipe as above for MM-Zn but with the use of dH_2_O, instead of Chelex® 100-treated (Bio-Rad) dH_2_O. For in vitro cultures, *C. neoformans* was pre-cultured in YPD media overnight, followed by subculture in yeast nitrogen base (YNB) medium with amino acids (BD Difco, Franklin Lakes, NJ) supplemented with 0.05% dextrose overnight, and sub-cultured in MM-Zn or MM + Zn and grown to mid-log phase. For macrophage infection, *C. neoformans* was grown overnight in YPD media at 37 °C, sub-cultured in YPD at 37 °C to mid-log phase. Samples were collected in triplicate for phenotypic and macrophage infection assays, and in quadruplicate for proteomic analyses.

### Sample preparation for mass spectrometry analysis

Sample preparation for mass spectrometry was performed as previously described [37]. Briefly, cell pellets were resuspended in 100 mM Tris-HCl (pH 8.5) and lysed using a probe sonicator (Thermo Fisher Scientific). Sodium dodecyl sulphate (SDS) and dithiothreitol (DTT) were added to final concentrations of 2% and 10 mM, respectively, followed by incubation at 95 °C for 10 min with shaking at 800 rpm, and incubation with 55 mM iodoacetamide (IAA) for 20 min in the dark. Next, ice cold 100% acetone was added to the samples to a final concentration of 80% and incubated overnight at − 20 °C. Samples were collected by centrifugation at 10,000 xg, 4 °C, for 10 min, washed with 80% acetone twice, air dried, and resuspended in 8 M urea/40 mM HEPES. Protein concentrations were determined using a bovine serum albumin (BSA) tryptophan assay [60]. Samples were diluted in 50 mM ammonium bicarbonate and normalized to 100 μg of protein prior to overnight digestion with a mixture LysC and trypsin proteases (Promega, protein:enzyme ratio, 50:1). To stop the digestion, 10% v/v trifluoroacetic acid (TFA) was added, and 50 μg of acidified peptides were desalted and purified using C18 (three layers) Stop And Go Extraction (STAGE) tips [38].

Secretome samples were processed according to an in-solution digestion as previously described [37]. Cellular debris was filtered from the culture supernatant by 0.22 μm syringe filters, then one-third volume of 8 M urea/40 mM HEPES was added to filtered supernatant followed by ultrasonication in ice bath for 15 cycles (30s on/30 s off). Samples were reduced and alkylated with DTT and IAA, respectively, followed by enzymatic digestion and STAGE-tip purification.

### Mass spectrometry

Mass spectrometry was performed as previously described with some modifications [37]. Purified peptides were lyophilized and resuspended in buffer A* (0.1% TFA) and analyzed by nanoflow liquid chromatography on an Ultimate 3000 LC system (Thermo Fisher Scientific) online coupled to QExactive HF quadrupole orbitrap mass spectrometer (Thermo Fisher Scientific). This includes a 5 mm μ-precolumn (Thermo Fisher Scientific) with 300 μm inner diameter filled with 5 μm C18 PepMap100 beads. Separation of peptides occurred on a 15 cm column with 75 μm inner diameter with 2 μm reverse-phase silica beads and directly electrosprayed into the mass spectrometer using a linear gradient from 4 to 30% ACN in 0.1% formic acid over 60 min at a constant flow of 300 nl/min. To clean the column, up to 95% ACN was used as washout following the linear gradient, and re-equilibrated to prepare the column for subsequent runs. The mass spectrometer was operated in data-dependent mode, switching automatically between one full scan and subsequent MS/MS scans of the fifteen most abundant peaks (Top15 method), with full scan (*m/z* 300–1650) acquired in the Oribtrap analyzer with a resolution of 60,000 at 100 *m/z*.

### Mass spectrometry data processing

Analysis of mass spectrometry raw data files were performed using MaxQuant software (version 1.6.0.26) [39]. The search was completed using the incorporated Andromeda search engine against the reference *C. neoformans* var. *grubii* serotype A (strain H99/ATCC 208821) proteome (Aug. 2018; 7,430 sequences) from Uniprot [61]. The parameters established include: trypsin enzyme specificity with 2 max missed cleavages; minimum peptide length of seven amino acids; fixed modifications – carbamidomethylation of cysteine, variable modifications – methionine oxidation and N-acetylation of proteins. Peptide spectral matches were filtered using a target-decoy approach at a false discovery rate (FDR) of 1% with a minimum of two peptides required for protein identification. Relative label-free quantification (LFQ) and match between runs was enabled with a match time window of 0.7 min, in which LFQ used the MaxLFQ algorithm integrated into MaxQuant using a minimum ratio count of one [62]. The. RAW and affiliated files were deposited into the publicly available PRIDE partner database for the ProteomeXchange consortium with the data set identifier: PXD023204.

### Bioinformatics

Statistical analysis and data visualization of the MaxQuant-processed data were performed using Perseus (version 1.6.2.2) [40]. Data were prepared by filtering proteins to the reverse database, contaminants, and proteins solely identified by one site, followed by log_2_ transformation of LFQ intensities. Identification of protein intensities present in triplicate within one sample set was filtered for statistical processing (3 valid values of 4 replicates in at least one group), missing values were imputed from normal distribution (width: 0.3, downshift: 1.8 standard deviations). Data corresponding to fold changes of growth conditions were identified using a Student’s *t*-test (*p* ≤ 0.05) with multiple hypothesis testing correction using the Benjamini-Hochberg FDR cut off at 0.05 [63]. Based on assessment of replicate reproducibility and protein identification numbers, one biological replicate from each condition in the cellular proteome data set was removed from further analysis. The projection of data was visualized with a principal component analysis (PCA) and hierarchical clustering (Pearson correlation) by Euclidean distance for replicate reproducibility. For 1D annotation enrichment, Student’s *t*-test (permutation-based FDR = 0.05; *S*_0_ = 1) was performed followed by 1D annotation enrichment function in Perseus using the Student’s *t*-test difference values with an FDR threshold of 0.05 using the Benjamini–Hochberg method. This analysis generates a numerical “score” value, which represents the direction in which the protein LFQ intensities within a given category tend to deviate from the overall distribution of all proteins. Visualization of 1D annotation enrichments by Gene Ontology and Keywords was performed within the RStudio platform (http://www.R-project.org/) [64]. The STRING functional protein association networks provided visualization of protein networks (https://string-db.org) [65].

### Construction of gene deletion and complementation strains

All gene deletion strains were produced by biolistic transformation of linear constructs generated by double joint PCR as previously described [66]. The constructs contained the nourseothricin (NAT) resistance cassette and were prepared with the reported primers and plasmids (Supp. Table 1). Each construct was generated by a first round of PCR amplification of the upstream (5′-gene) and downstream (3′-gene) regions of *WOS2* using primers P1/P3 and P4/P6. The NAT resistance marker was amplified from pAI3 (generously provided by Dr. J. P. Xu, McMaster University) using primers NAT F/R. The 5′-gene and NAT amplicon were combined by a double-joint PCR with primers P1/P10, followed by similarly linking the 3′-gene and NAT amplicons with primers P9/P6. The amplified deletion cassette purified using the QIAquick Gel Extraction Kit (Qiagen) and was combined with gold microcarrier beads (Bio-Rad) and introduced into *C. neoformans* H99 strain via biolistic transformation [67]. Stable transformants (i.e., two independent mutants) were selected on YPD-NAT (100 μg/mL) plates and confirmed by diagnostic PCR, multiple stable mutants were constructed in independent transformation experiments. Insertion of NAT into a single site within the *C. neoformans* genome was confirmed by whole genome assembly (Supp. Fig. 4).

To construct the Wos2-FLAG complemented strain, the *WOS2* open reading frame (ORF) and promoter region were amplified with 7558_NOTI_Promoter_F/7558_NOTI_R. The amplified PCR product was digested using NotI and cloned into plasmid pHP1, which contains a 4X FLAG tag and hygromycin selectable marker (generously provided by Dr. J. Heitman, Duke University) [68]. Correct insert orientation for a 3′ gene FLAG-tag was confirmed using primer pair M13_F/7558_pHP1_R, followed by sequencing of the constructed site at the Advanced Analysis Centre – Genome facility (University of Guelph). The resulting *WOS2*-FLAG plasmid was introduced into the *wos2*Δ strain using biolistic transformation, and multiple transformants were selected using YPD supplemented with hygromycin B (100 μg/mL) (Sigma-Aldrich) and were confirmed by western blot (Supp. Fig. 5).

For Western blotting, whole cell extracts of the *C. neoformans* strains were separated by SDS-PAGE and transferred to a polyvinylidene difluoride membrane using a transfer apparatus, according to the manufacturer’s protocols (Bio-Rad), as previously described [22]. Briefly, membranes were blocked with 3% non-fat milk in 1X TBS (50 mM Tris, 150 mM NaCl, pH 7.5) at 4 °C overnight, followed by washing with TBST (1X TBS, 0.05% Tween-20) five times. Next, membranes were incubated with Monoclonal ANTI-FLAG® M2 antibody (Sigma-Aldrich) for 1 h, washed three times for 5 min, and incubated with 1:3000 dilution of horseradish peroxidase-conjugated Goat anti-mouse IgG Fc secondary antibody (Invitrogen) for 1 h. Incubations were performed at room temperature. Blots were washed three times with TBST and developed using the Clarity Max Western ECL Substrates system (BioRad). The xxperiment was performed in biological and technical duplicates.

### Genomic DNA extraction, Illumina sequencing and whole genome analysis

Genomic DNA (gDNA) was extracted from *C. neoformans* H99 WT and independent *wos2*Δ mutants using PureLink™ Genomic DNA Mini Kit (ThermoFisher Scientific). Purified gDNA were prepared using an Illumina Nextera kit by the Microbial Genome Sequencing Center (Pennsylvania, USA) followed by Illumina sequencing on a NextSeq 550 platform. Raw .fastq files were processed using Geneious Prime 2021.0 (www.geneious.com) [69]. *C. neoformans* H99 WT files were initially trimmed using an in-suite BBDuk plug-in to trim adaptors and low-quality reads [70]. The trimmed reads were mapped to a reference genome retrieved from NCBI. Correct assembly was validated by searching for variations (i.e., deletion or insertion) and SNPs. Upon confirmation of correct assembly, the independent deletion mutants were mapped to the assembled WT genome. To identify any non-specific insertions of NAT into the *C. neoformans* mapping, settings were modified to search for insertions or deletions up to 1600 bp (matching the expected size of the NAT gene). Whole genome alignment between the WT and *wos2*Δ strain was performed using a progressiveMauve alignment from the Mauve plug-in using default alignment settings for a single chromosome and Mauve Contig Mover algorithm ordered and aligned 15 contigs (i.e., 14 chromosomes and one mitochondrion) [71, 72].

### Growth curves and zinc utilization

To analyse thermotolerance of *C. neoformans* strains, fungal cells were grown overnight at 30 °C in YPD, followed by 1:100 subculture in YNB. For growth curves, resuspended fungal cells inoculated into a final volume of 200 μL with 1 × 10^5^ cells/mL into zinc-replete media and incubated at 30 °C or 37 °C. OD_600nm_ measurements were performed on BioTek HM1 plate reader every 15 min over 80 h. For zinc utilization assays, cells were collected by centrifugation at 1500 x g for 5 mins, washed twice with MM-Zn and resuspended in MM-Zn or MM + Zn followed by serial dilution in tenfold (10^6^ cells/ml) on zinc-limited or -replete agar. Plates were incubated at either 30 °C or 37 °C for 3 d, with images taken every 24 h.

### Capsule assay and shedding

To visualize polysaccharide capsule production, *C. neoformans* strains were grown overnight at 37 °C in YPD, followed by 1:100 subculture in YNB overnight, cells were washed twice in LIM, and inoculated into LIM for 16 h at 37 °C [73]. Capsule production was examined by differential interference contrast microscopy by staining with India ink dye (Hardy Diagnostics). Cell diameter and capsule thickness were measured for 50 cells per strain, and relative capsule sizes were defined as the ratio of the capsule thickness to the diameter of the cell using ImageJ software (https://imagej.nih.gov/ij/index.html). For the capsule shedding blot, supernatant was collected from each *C. neoformans* strain following 72 h of growth in LIM and diluted to an OD_600nm_ of 1 [74]. The supernatant was denatured at 70 °C for 15 min, run on a 0.6% agarose gel, and blotted onto nylon membrane (GE healthcare), followed by membrane incubation with a 1:1000 dilution of 18B7 monoclonal antibody. Next, a 1:2500 dilution of anti-mouse horseradish peroxidase antibody was added, and polysaccharide was visualized by chemiluminescence (GE Healthcare) (Supp. Fig. 6).

### Melanin plate assay

To examine the effects of gene deletion on *C. neoformans* ability to produce melanin pigmentation, *C. neoformans* strains were grown overnight at 30 °C in YPD, followed by 1:100 subculture and overnight incubation in YNB. Next, cultures underwent a final 1:100 overnight subculture into MM, washed with 1 mL of MM, and serially diluted tenfold (10^6^ cells/ml) on MM agar containing 1 mM L-DOPA (Sigma-Aldrich) and incubated at either 30 °C or 37 °C for 7 days, with imaging every 24 h.

### Macrophage infection

Immortalized murine macrophages originally derived from WT BALB/c mice (generously provided by Dr. Felix Meissner, Max Planck) were maintained at 37 °C in 5% CO_2_ in Dulbecco’s Modified Eagle’s Medium (DMEM) supplemented with 10% heat-inactivated fetal bovine serum (FBS; ThermoFisher), 2 mM Glutamax, 1% sodium pyruvate, 1% L-glutamine, and 5% penicillin/streptomycin. Macrophages were seeded in 12-well plates at 0.1 × 10^6^ cells/well and grown until 70–80% confluence was reached (approx. 48 h). *C. neoformans* strains were grown to mid-log phase in YPD at 37 °C, collected at 1500 xg for 10 min, washed twice in PBS, and resuspended in DMEM without pen/strep. Fungal cells were opsonized with mAb18B7 (1 μg: 10^6^ fungal cells) for 1 h at 37 °C and 5% CO_2_ [75, 76]. Macrophage were infected at a multiplicity of infection (MOI) of 10:1 (cryptococcal cells:macrophages) for 90 min at 37 °C, 5% CO_2_. Following co-culture, cells were washed with PBS to remove unattached and extracellular fungal cells, and fresh DMEM without pen/strep was added for the remainder of the assay.

### Cytotoxicity assays

Quantification of macrophage death was performed as previously described with the following modifications [16]. The culture supernatants of *C. neoformans*-infected BALB/c macrophages were collected at 3, 20, 24, 40, and 48 h post-inoculation (hpi) and the percentage of cell death was quantified with the CytoTox96® Non-Radioactive Cytotoxicity Assay (Promega) according to manufacturer’s instructions. Quantification of cell death was performed in biological triplicate, and the experiment was performed in technical duplicate.

## Supplementary Information

**Additional file 1.**

**Additional file 2.**

**Additional file 3.**

**Additional file 4.**

**Additional file 5.**

**Additional file 6.**

**Additional file 7.**

## Data Availability

The. RAW and affiliated files were deposited into the publicly available PRIDE partner database for the ProteomeXchange consortium with the data set identifier: PXD023204. Reviewer login: reviewer_pxd023204@ebi.ac.uk; password: 31XS7ipW.
