# Supplementary material for: Proteome and secretome profiling of zinc availability in Cryptococcus neoformans identifies Wos2 as a subtle influencer of fungal virulence determinants
Source: BMC Microbiol. 2021 Dec 13;21:341. doi: 10.1186/s12866-021-02410-z (PMC8667453; doi:10.1186/s12866-021-02410-z)
Supplement: Supplementary file 2 — Additional file 2. [file 12866_2021_2410_MOESM2_ESM.docx]

**Title: Proteome and secretome profiling of zinc availability in *Cryptococcus neoformans* identifies Wos2 as a subtle influencer of fungal virulence determinants**

**Authors:** Ball, B., Woroszchuk, E., Sukumaran, A., West, H., Afaq, A., Carruthers-Lay, D., Muselius, B., Gee, L., Langille, M., Pladwig, S., Kazi, S., Hendriks, A., Geddes-McAlister, J.*

**Supp. Fig. 2: Replicate reproducibility of *Cryptococcus neoformans* secretome under regulated zinc levels.** Column correlation by Pearson correlation was performed and mapped by Euclidean distance. Replicate reproducibility varied between 83.6 – 89.4%.
