# Supplementary material for: Proteome and secretome profiling of zinc availability in Cryptococcus neoformans identifies Wos2 as a subtle influencer of fungal virulence determinants
Source: BMC Microbiol. 2021 Dec 13;21:341. doi: 10.1186/s12866-021-02410-z (PMC8667453; doi:10.1186/s12866-021-02410-z)
Supplement: Supplementary file 3 — Additional file 3. [file 12866_2021_2410_MOESM3_ESM.docx]

**Title: Proteome and secretome profiling of zinc availability in *Cryptococcus neoformans* identifies Wos2 as a subtle influencer of fungal virulence determinants**

**Authors:** Ball, B., Woroszchuk, E., Sukumaran, A., West, H., Afaq, A., Carruthers-Lay, D., Muselius, B., Gee, L., Langille, M., Pladwig, S., Kazi, S., Hendriks, A., Geddes-McAlister, J.*


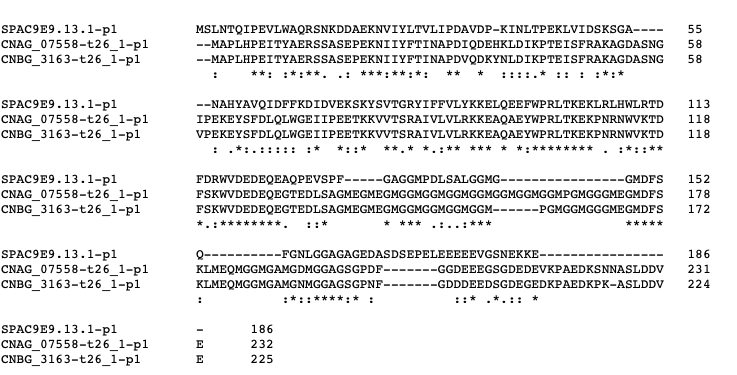


**Supp. Fig. 3: Multiple sequence alignments for Wos2.** Clustal O (operated through FungiDB) was used to align sequences of orthologues for CNAG_07558 (Wos2). *Cryptococcus neoformans* CNAG_07558 (CNAG_07558-t26_1-p1) was mapped to *Cryptococcus gattii* (CNBG_3163-t26_1-p1, and *Schizosaccharomyces pombe* (SPAC9E9.13.1-p1).
