# Supplementary material for: Proteome and secretome profiling of zinc availability in Cryptococcus neoformans identifies Wos2 as a subtle influencer of fungal virulence determinants
Source: BMC Microbiol. 2021 Dec 13;21:341. doi: 10.1186/s12866-021-02410-z (PMC8667453; doi:10.1186/s12866-021-02410-z)
Supplement: Supplementary file 4 — Additional file 4. [file 12866_2021_2410_MOESM4_ESM.docx]

**Title: Proteome and secretome profiling of zinc availability in *Cryptococcus neoformans* identifies Wos2 as a subtle influencer of fungal virulence determinants**

**Authors:** Ball, B., Woroszchuk, E., Sukumaran, A., West, H., Afaq, A., Carruthers-Lay, D., Muselius, B., Gee, L., Langille, M., Pladwig, S., Kazi, S., Hendriks, A., Geddes-McAlister, J.*


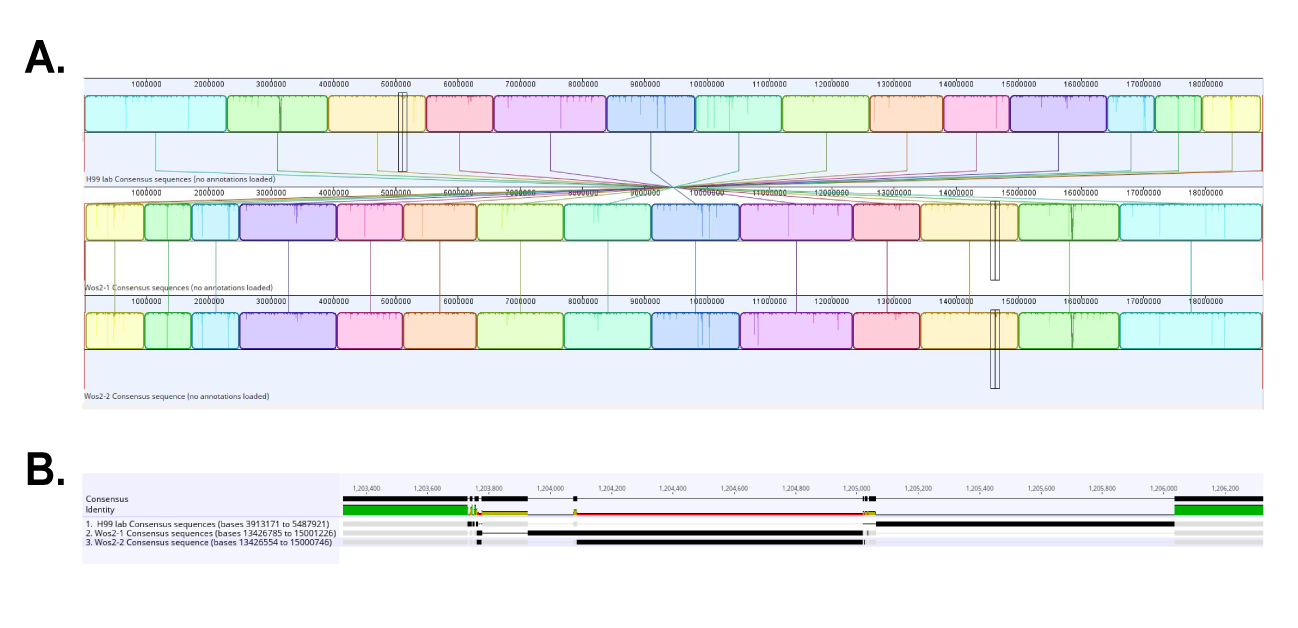


**Supp. Fig. 4:** **Mauve alignment of the whole genome sequences of wildtype *C. neoformans* H99 and *wos2*Δ deletion strain**. **A)** Mauve Contig Mover (MCM) algorithm ordered and aligned 15 contigs (i.e., 14 chromosomes and one mitochondrion) of *wos2*Δ strain to the reference sequence of *C. neoformans* H99, resulting in 18.9 Mb identical sites with 99.9% pairwise identity. **B)** ProgressiveMauve algorithm aligned chromosome three, containing the targeted gene deletion, resulting in 1.57 Mb identical sites and a 99.8% pairwise identity. Highlighted region of H99 and *wos2*Δ alignment featuring a deletion of *WOS2* and insertion of the nourseothricin selectable marker (1,203,529-1,205,882 bp position).
