# Supplementary material for: Proteome and secretome profiling of zinc availability in Cryptococcus neoformans identifies Wos2 as a subtle influencer of fungal virulence determinants
Source: BMC Microbiol. 2021 Dec 13;21:341. doi: 10.1186/s12866-021-02410-z (PMC8667453; doi:10.1186/s12866-021-02410-z)
Supplement: Supplementary file 5 — Additional file 5. [file 12866_2021_2410_MOESM5_ESM.docx]

**Title: Proteome and secretome profiling of zinc availability in *Cryptococcus neoformans* identifies Wos2 as a subtle influencer of fungal virulence determinants**

**Authors:** Ball, B., Woroszchuk, E., Sukumaran, A., West, H., Afaq, A., Carruthers-Lay, D., Muselius, B., Gee, L., Langille, M., Pladwig, S., Kazi, S., Hendriks, A., Geddes-McAlister, J.*


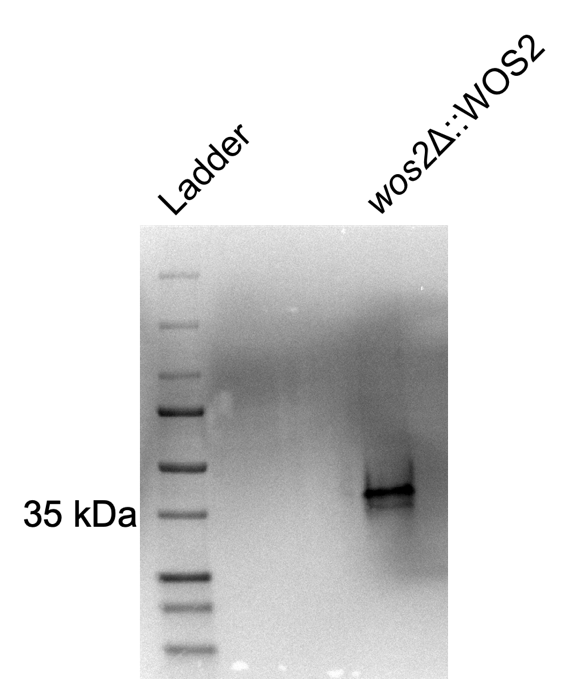


**Supp. Fig. 5: Western blot of whole cell extracts to confirm complementation of *WOS2*.** SDS-PAGE transferred to polyvinylidene difluoride membrane followed by incubation with Monoclonal ANTI-FLAG® M2 antibody. Wos2 with FLAG tag is approx. 31 kDa.
