# Supplementary material for: Proteome and secretome profiling of zinc availability in Cryptococcus neoformans identifies Wos2 as a subtle influencer of fungal virulence determinants
Source: BMC Microbiol. 2021 Dec 13;21:341. doi: 10.1186/s12866-021-02410-z (PMC8667453; doi:10.1186/s12866-021-02410-z)
Supplement: Supplementary file 6 — Additional file 6. [file 12866_2021_2410_MOESM6_ESM.docx]

**Title: Proteome and secretome profiling of zinc availability in *Cryptococcus neoformans* identifies Wos2 as a subtle influencer of fungal virulence determinants**

**Authors:** Ball, B., Woroszchuk, E., Sukumaran, A., West, H., Afaq, A., Carruthers-Lay, D., Muselius, B., Gee, L., Langille, M., Pladwig, S., Kazi, S., Hendriks, A., Geddes-McAlister, J.*


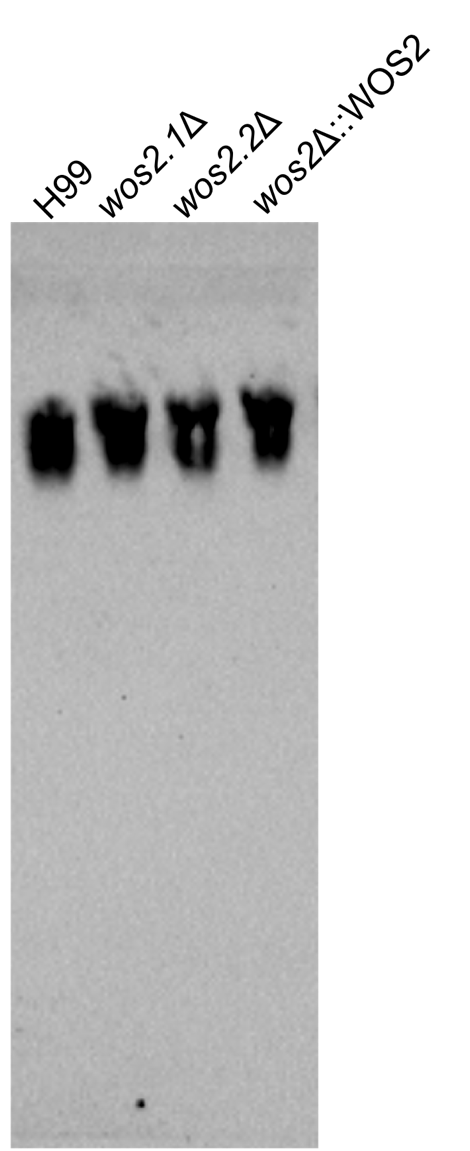


**Supp. Fig. 6: Capsule shedding blot for *C. neoformans* strains.** Supernatant was collected from each strain following 72 h in low iron media followed by agarose gel, blotting, and incubations with the 18B7 monoclonal antibody.
